# Survival status and predictors of mortality among neonates admitted to neonatal intensive care unit at Bichena Primary Hospital, Northwest Ethiopia. A retrospective cohort study

**DOI:** 10.3389/fped.2025.1529089

**Published:** 2025-04-09

**Authors:** Anley Shiferaw Enawgaw, Debas Belay, Alehegn Nigate, Almaw Genet Yeshiwas, Tesfaye Shumet, Bekalu Endalew, Keralem Anteneh Bishaw

**Affiliations:** ^1^ Department of Public Health, College of Health Sciences, Debre Markos University, Debre Markos, Ethiopia; ^2^ Department of Public Health, Bichena Primary Hospital, Bichena, Amhara, Ethiopia; ^3^ Department of Nursing, Bichena Primary Hospital, Bichena, Amhara, Ethiopia; ^4^ Department of Environmental Health, Injibara University, Injibara, Amhara, Ethiopia; ^5^ Department of Midwifery, College of Health Sciences, Debre Markos University, Debre Markos, Ethiopia

**Keywords:** survival status, predictors, neonate, primary hospitals, Ethiopia

## Abstract

**Background:**

Despite progress in reducing neonatal mortality rates in Ethiopia, the country still has a high neonatal mortality rate compared with the global average. Primary hospitals are critical in delivering basic neonatal care, particularly in rural areas. However, data on neonatal mortality and contributing factors in these settings are scarce. This study aimed to determine the survival status and predictors of neonatal mortality among neonates admitted to Bichena Primary Hospital, Northwest Ethiopia.

**Methods:**

A retrospective cohort study was conducted among 638 neonates admitted to the Bichena Primary Hospital neonatal intensive care unit from January 1, 2021, to April 30, 2023. Neonates were selected via a consecutive sampling method. Data were collected from medical records using a pretested checklist. A Kaplan–Meier survival curve was used to estimate the neonatal survival time, and a Cox proportional hazard regression model was used to identify independent predictors of neonatal mortality.

**Results:**

Of the 638 neonates followed, 21.5% died during the study period. The overall incidence rate of death was 66.69 per 1,000 neonate days. Hypothermia, birth injury, perinatal asphyxia, preterm birth, maternal history of abortion, low birth weight, and neonatal hypoglycemia were independent predictors of neonatal mortality.

**Conclusion and recommendation:**

The study found a high rate of neonatal mortality, exceeding rates reported in other regions of Ethiopia. Most predictors were preventable and treatable. Therefore, early identification of obstetric complications, immediate interventions and postnatal care are crucial to reduce neonatal mortality and enhance overall neonatal outcomes.

## 1 Introduction

### 1.1 Background

Neonatal mortality refers to the death of a live-born baby within the first 28 days (1) and measures the quality of maternal and child healthcare (2). Neonatal deaths account for 47% of the global under-five mortality (3). Among these deaths, 36% occur during the first 24 h (2, 4), 37% occur between days 1 and 7, and 27% occur between days 7 and 27 (2). In low- and middle-income countries, neonatal deaths account for 53.1% of all under5 deaths (5). From 1990 to 2019, neonatal deaths worldwide decreased from 5 million to 2.4 million (3), resulting in a decline in the mortality rate from 37 deaths per 1,000 live births in 1990 to 18 deaths per 1,000 live births in 2021 (6).

In East Africa, the Neonatal mortality rate (NMR)increased from 33.6 per 1,000 live births in 2017 (7) to 51.32 per 1,000 live births in 2021 (8). NMRs also vary across countries: Kenya reported a rate of 19.6 per 1,000 live births in 2018 (9), Eritrea reported a rate of 16.5 (10), and Ethiopia reported 26.84 per 1,000 live births (11). In Ethiopia, neonatal mortality is a major public health issue (12). The Amhara region has the highest rate of 46 per 1,000 live births, which is higher than that reported in the other areas (12). There is also a geographical clustering of neonatal deaths in Ethiopia, with Amhara being one of the most affected areas (7, 13), emphasizing the need to address NMRs in this region.

Neonatal mortality in Ethiopia improved from 39 deaths per 1,000 live births in 2005–29 deaths in 2016 (14) but slightly increased to 33 deaths in 2019 (12), which is higher than the global average of 18 deaths per 1,000 live births (4). This suggests inadequate progress toward achieving Sustainable Development Goals (SDG) 3.3, which aims to reduce NMRs to less than 12 by 2030. The identified risk factors were home birth, rural residency, gestational age, sepsis, perinatal asphyxia (PNA), longer birth intervals, and respiratory distress (7, 15). Additionally, mothers of advanced age and primipara mothers are at increased risk of neonatal mortality (11).

Numerous efforts are underway to improve neonatal survival. The SDGs aim to reduce neonatal mortality to 12 per 1,000 live births by 2030 (4). Additionally, the Every Newborn Action Plan (ENAP) focuses on preventing stillbirths and newborn deaths (16). Ethiopia has committed to integrating life-saving measures into the national strategy outlined in the Health Sector Transform Plan (HSTP), to achieve an NMR of 10 per 1,000 live births by 2020 (17).

Despite efforts, neonatal mortality remains high in Ethiopia, with a current rate of 33 deaths per 1,000 live births (14). To reduce NMRs, it is crucial to identify and address the underlying causes of neonatal deaths starting from primary hospitals, which are the first point of care for newborns (18), and provide basic neonatal care (19). Therefore, this study aimed to investigate the survival status and predictors of mortality among neonates admitted to Bichena Primary Hospital in Northwest Ethiopia. These findings support the goals of the Ethiopian Hospital Alliance for Quality (EHAQ) program by generating new evidence to support evidence-based care in neonatal care (20).

## 2 Methods and materials

### 2.1 Study area and period

The study took place at the Bichena Primary Hospital neonatal intensive care unit located in Bichena Administrative Town in the Amhara Region, Northwest Ethiopia. It is approximately 273 km from Addis Ababa, the capital of Ethiopia, and 225 km from Bahir Dar, the capital of the Amhara Regional State. The hospital has been operational since April 2015, providing healthcare services to a population exceeding 450,000. In addition to providing various inpatient and outpatient services, including a neonatal intensive care unit, the hospital provides to the areas surrounding Enemay Woreda and Debay Telatgen.

The neonatal intensive care unit of Bichena Primary Hospital has a maximum of 9 beds, with a monthly average of 35 neonates being admitted, yielding, on average, 400–500 annual admissions of neonates. During the study period from January 1, 2021, to April 2023, the NICU was equipped with four room heaters, one infant radiant warmer, one fan radiant warmer, one incubator, four oxygen concentrators, and oxygen cylinders. However, Bubble CPAP was not available.

### 2.2 Study design

A retrospective cohort study was conducted among neonates admitted to the NICU (Neonatal Intensive Care Unit) at Bichena Primary Hospital, Northwest Ethiopia.

### 2.3 Populations

#### 2.3.1 Source population

The source population comprised all neonates admitted to the NICU at Bichena Primary Hospital, North West Ethiopia, during the study period.

#### 2.3.2 Study population

The study population comprised neonates admitted to the NICU at Bichena Primary Hospital, Northwest Ethiopia, between January 1, 2021, and April 2023, who met the inclusion criteria.

### 2.4 Eligibility criteria

#### 2.4.1 Inclusion criteria

All admitted neonates’ medical records with complete information during the study period (January 1, 2021–April 30, 2023) in the NICU at Bichena Primary Hospital were included.

#### 2.4.2 Exclusion criteria

Neonates with incomplete records of major variables (e.g., date of admission, date of discharge/death, treatment outcomes), lost records, revisits, or neonates who were initially admitted but were immediately referred to specialized health facilities for further management were excluded from the study.

### 2.5 Sample size determination and sampling procedure

#### 2.5.1 Sample size determination

The sample size is estimated via the double population proportion formula in Epi Info version 7. This is done by considering different significant variables from the previous study and by considering the following assumption: the level of significance is 5%, the power is 80%, P1 is the proportion of neonatal mortality among the exposed group, and P2 is the proportion outcome among the not exposed group. The ratio of the population exposed to not exposed neonates was 1:1. The anticipated mortality rate among neonates who were delivered via assisted methods (P1, exposed) was 12.2%, whereas that for those delivered without assistance (P2, unexposed) was 21.2% (21); 10% was added for potential incompleteness. Hence, the final sample size was determined to be 638 neonates admitted to the NICU at Bichena Primary Hospital.

#### 2.5.2 Sampling technique and procedures

A consecutive sampling method was employed to select study participants, involving the sequential selection of participants using their medical record numbers until the desired sample size was achieved.

### 2.6 Study variables

The dependent variable for this study was the time to death of the neonate. The independent variables were sociodemographic factors, including maternal age, place of residence, sex of the neonate, and age of the neonate; maternal and obstetric factors, gravidity, parity, Antenatal care (ANC), mode of delivery, place of delivery, duration of labor, time of initiation of breastfeeding, amniotic fluid status, premature rupture of membrane (PROM), antepartum hemorrhage, eclampsia, type of pregnancy, history of abortion, stillbirth, history of fetal death, anemia, hepatitis, Syphilis and HIV/AIDS; and neonatal factors, including date of admission, weight, Apgar (Appearance, Pulse, Grimace, Activity, Respiration) score, and gestational age. Neonatal medical conditions such as sepsis, perinatal asphyxia (PNA), respiratory distress syndrome (RDS), sepsis, hypoglycemia, and temperature at admission.

### 2.7 Operational definitions

Neonatal anemia was defined as a hemoglobin level of less than 13 gr/dl or a hematocrit of less than 39% (22).

Birth asphyxia was diagnosed when the newborn had at least one of the following signs: not breathing or gasping, <30 breaths per minute, or an APGAR score <7, neonatal neurologic sequelae (seizures, coma, and hypotonic), or multiple organ involvement (kidney, lungs, liver, heart, and intestines) (23).

Censored: a neonate who was alive at the end of the study or lost to follow-up, including discharged to home, discharged against medical advice, or transferred to other health institutions without knowing the outcome (24).

Event: death of a neonate after NICU admission as evidenced by physician confirmation.

Follow-up time-From the time of admission until either an event or censorship occurs.

Hepatitis: Diagnosed as positive if the hepatitis antigen test is positive during pregnancy or during neonate admission if not tested previously.

Low birth weight: defined as a birth weight admission weight of less than 2,500 grams.

Preterm: refers to births occurring before 37 completed weeks of gestation (25).

Survival status: outcome of the neonate; either death or censored.

Survival time: Measures the follow-up time from admission to the NICU to the occurrence of an event or censoring (26).

Syphilis: Diagnosed as positive if the VDRL test is positive during pregnancy or during neonate admission if not tested previously.

Time origin: date of admission.

Time scale: days from the admission of a neonate to the occurrence of an outcome.

### 2.8 Data collection tools and procedures

The data extraction tool was adapted from the NICU registration book (27), which has been used by other studies previously (26, 28–30). The questionnaire was prepared in the English language. Four BSC nurses currently working in the NICU of Bichena Primary Hospital were involved as data collectors, and two MSC nurses who are hospital staff were supervisors. Survival time was obtained by considering the time between the date of admission and the date of discharge (the date of death or censoring).

### 2.9 Data quality control

A pretest was performed on 5% (32) of the sample size in the hospital before the actual data were collected. The necessary modifications and corrections were made to ensure its validity. Before starting the data collection, one-day training was given on the objectives of the study, the contents of the checklist, and issues related to the confidentiality of the neonates’ profiles. The principal investigator and supervisors spot-checked and reviewed the completed checklist to ensure completeness and were examined through a random selection of cards by the principal investigator.

### 2.10 Data processing and analysis

The data were cleaned, coded, and edited to ensure completeness and consistency. The cleaned data were entered into Epi-Data version 3.1 and exported to STATA 14.0 for analysis.

Descriptive statistics, such as frequencies with percentages and means with standard deviations, were used to describe the study population. Tables and figures were also used for data presentation. A variance inflation factor (VIF) >10 and tolerance (T) <0.1 were considered suggestive of the existence of multicollinearity. We found that none of the variables in our model had a VIF greater than 10 or a T less than 0.1, indicating that multicollinearity was not a concern.

A Kaplan–Meier curve was used to estimate the survival status of neonates admitted to the NICU at different times. The log-rank test was used to compare the survival experience of categorical predictor variables.

The assumptions of proportional hazards were verified via statistical tests and graphical tests. The log minus log plots were assessed to confirm whether they were approximately parallel to each other over time, indicating that the proportional hazards assumption was met. The *p* values of all variables and the overall global test results in the final multivariable Cox regression model were assessed, with values greater than 0.05 indicating that the proportional hazard assumption was fulfilled. Schoenfeld residual proportional hazard test was used to confirm the independence between the residuals and time. A slope of zero between the residuals and time indicated a good model fit. The Cox-Snell residuals test was also used to evaluate the model's goodness of fit.

Factors associated with outcome variable with a *p*-value less than 0.25 in bivariate Cox regression were analyzed via multivariate Cox regression. The adjusted hazard ratios (AHR) with 95% confidence intervals (CIs) from the multivariate Cox regression were used as a measure of association, and variables with a *p*-value <0.05 were considered significant predictors.

## 3 Results

### 3.1 Sociodemographic characteristics of the study participants

A total of 638 neonates were followed during the study period. Among the participants, the majority of neonates in the study were male (61.44%), while the percentage of deaths was slightly greater in females (22.76%) than in males (20.66%). Furthermore, a notable 72.1% of neonates were admitted within 24 h of birth. Concerning residence, approximately half (55.96%) of the neonates were from urban areas. Additionally, the majority of mothers (42.32%) were in the age range of 25–34 years. Finally, 61.6% of the newborns were born at term, whereas 38.4% were born preterm (Table 1).

**Table 1:** Sociodemographic characteristics of neonates and their mothers in study.

Variables	Outcome status		Total frequency (percentage)
	Censored (%)	Dead (%)	
Sex			
Male	311 (79.34)	81 (20.66)	392 (61.44)
Female	190 (77.24)	56 (22.76)	246 (38.56)
Age at admission			
Less than 24 h	350 (76.09)	110 (23.91)	460 (72.1)
Greater than 24 h	151 (84.83)	27 (15.17)	178 (27.9)
Residence			
Urban	291 (81.51)	66 (18.49)	357 (55.96)
Rural	210 (74.73)	71 (25.27)	281 (44.04)
Maternal age			
<=20 years	48 (70.59)	20 (29.41)	68 (10.66)
20–24 years	116 (82.86)	24 (17.14)	140 (21.94)
25–34 years	221 (81.85)	49 (18.15)	270 (42.32)
>=35 years	41 (73.21)	15 (26.79)	56 (8.78)
Not documented	75 (72.12)	29 (27.88)	104 (16.3)
Gestational age			
Preterm	176 (71.84)	69 (28.16)	245 (38.4)
Term	325 (82.70)	68 (17.30)	393 (61.6)
Amission weight			
Normal	275 (83.59)	54 (16.41)	329 (51.57)
Low	226(73.14)	83(26.86)	309(48.43)

### 3.2 Maternal and obstetric characteristics of mothers

Among the 638 neonates in the study, the majority of mothers (94.51%) received ANC, whereas 5.49% did not. The majority of neonates were born spontaneously (66.77%), followed by cesarean section (12.7%), instrumental delivery (17.71%), and assisted breech delivery (2.82%). The place of delivery varied, with 63.48% of neonates born at the same facility, 29.31% from other facilities, and 7.21% born at home. Regarding the history of abortion, 97.18% of the neonates were born to mothers with no history of abortion. The majority of neonates (88.71%) were born without any obstetric complications, although 11.29% experienced complications (Table 2).

**Table 2:** Maternal and obstetric characteristics of mothers whose neonates were admitted in the study.

Variables	Outcome status		Total frequency (percentage)
	Censored (%)	Dead (%)	
Mode of delivery			
Spontaneous	336 (78.87)	90 (21.13)	426 (66.77)
CS^a^	61 (75.31)	20 (24.69)	81 (12.7)
Instrumental	93 (82.3)	20 (17.7)	113 (17.71)
Assisted breech	11 (61.11)	7 (38.89)	18 (2.82)
Place of delivery			
Home delivery	37 (80.43)	9 (19.57)	46 (7.21)
Same facility	317 (78.27)	88 (21.73)	405 (63.48)
Referred from other	147 (78.61)	40 (21.39)	187 (29.31)
Gravidity			
Primigravida	242 (81.21)	56 (18.79)	298 (46.71)
Multigravida	259 (76.18)	81 (23.82)	340 (53.29)
Premature PROM^a^			
Yes	35 (72.92)	13 (27.08)	48 (7.52)
No	466 (78.98)	124 (21.02)	590 (92.48)
Prolonged PROM^a^			
No	484 (79.21)	127 (20.79)	611 (95.77)
Yes	17 (62.96)	10 (37.04)	27 (4.23)
Parity			
Para I	246 (77.36)	72 (22.64)	318 (49.84)
Multi	217 (81.27)	50 (18.73)	267 (41.85)
Grand Para	38 (71.70)	15 (28.30)	53 (8.31)
History of abortion			
No	493 (79.52)	127 (20.48)	620 (97.18)
Yes	8 (44.44)	10 (55.56)	18 (2.82)
History of stillbirth			
No	494 (78.54)	135 (21.46)	629 (98.59)
Yes	7 (77.78)	2 (22.22)	9 (1.41)
History of fetal death			
No	480 (78.82)	129 (21.18)	609 (95.45)
Yes	21 (72.41)	8 (27.59)	29 (4.55)
Obstetric complications			
No	444 (78.45)	122 (21.55)	566 (88.71)
Yes	57 (79.17)	15 (20.83)	72 (11.29)
CPD^a^			
Yes	7 (77.78)	2 (22.22)	9 (1.41)
No	494 (78.54)	135 (21.46)	629 (98.59)
Antepartum hemorrhage			
Yes	4 (66.67)	2 (33.33)	6 (0.94)
No	497 (78.64)	135 (21.36)	632 (99.06)
ANC^a^			
No	29(82.86)	6(17.14)	35(5.49)
Yes	472(78.28)	131(21.72)	603(94.51)

^a^ CS, cesarean section; PROM, premature rupture of membrane; CPD, cephalopelvic disproportion; ANC, antenatal care.

### 3.3 Maternal medical diagnosis

In the present study, among mothers of neonates, 2.51% were HIV positive, whereas 97.49% were not exposed. Hepatitis was present in a small fraction (1.41%) of mothers. Eclampsia was diagnosed in 3.13% of the mothers of the neonates (Table 3).

**Table 3:** Maternal medical diagnoses of neonates admitted to the NICU in the study.

Variables	Outcome status		Total frequency (percentage)
	Censored (%)	Dead (%)	
HIV^a^			
Positive	11 (68.75)	5 (31.25)	16 (2.51)
Negative	490 (78.78)	132 (21.22)	622 (97.49)
Hepatitis			
Positive	6 (66.67)	3 (33.33)	9 (1.41)
Negative	495 (78.70)	134 (21.30)	629 (98.59)
Syphilis			
Positive	9 (75.00)	3 (25.00)	12 (1.88)
Negative	492 (78.59)	134 (21.41)	626 (98.12)
Any medical diagnosis			
No	487 (78.8)	131 (21.2)	618 (96.87)
Yes	14 (70)	6 (30)	20 (3.13)
Eclampsia			
Yes	17 (85)	3 (15)	20 (3.13)
No	484 (78.32)	134(21.68)	618(96.87)

^a^ HIV, human immunodeficiency virus.

### 3.4 Neonatal medical diagnosis

Among the admitted neonates, the majority (86.05%) were hypothermic during admission. Neonates admitted with suspected neonatal sepsis accounted for 67.55% of the neonates. Almost three-fourths (74.29%) of the neonates were asphyxiated, and 18.03% of them received resuscitation. Birth injury or trauma was observed in 18.97% of the neonates (Table 4).

**Table 4:** Neonatal medical-related factors among neonates admitted to the NICU of Bichena primary hospital Northwest Ethiopia, January 1st, 2021–April 30, 2023.

Variables	Outcome status		Total frequency (percentage)
	Censored (%)	Dead (%)	
5th minute APGAR^a^			
Normal apgar	280 (79.1)	74 (20.9)	354
Low apgar	109 (72.67)	41 (27.33)	150
Not documented	112 (83.58)	22 (16.42)	134
PNA^a^			
No	396 (83.54)	78 (16.46)	474 (74.29)
Yes	105 (64.02)	59 (35.98)	164 (25.71)
Resuscitation			
No	424 (81.07)	99 (18.93)	523 (81.97)
Yes	77 (66.96)	38 (33.04)	115 (18.03)
Twin			
No	440 (79.28)	115 (20.72)	555 (86.99)
Yes	61 (73.49)	22 (26.51)	83 (13.01)
RDS^a^			
Yes	59 (68.60)	27 (31.40)	86 (13.48)
No	442 (80.07)	110 (19.93)	552 (86.52)
Suspected sepsis			
Yes	327 (75.87)	104 (24.13)	431 (67.55)
No	174 (84.06)	33 (15.94)	207 (32.45)
Amniotic fluid status			
Clear	417 (80.81)	99 (19.19)	516 (80.88)
Meconium stained	84 (68.85)	38 (31.15)	122 (19.12)
Hypothermia			
Yes	419 (76.32)	130 (23.68)	549 (86.05)
No	82 (92.13)	7 (7.87)	89 (13.95)
Hypoglycemia			
No	461 (80.03)	115 (19.97)	576 (90.28)
Yes	40 (64.52)	22 (35.48)	62 (9.72)
Birth injury/trauma			
No	419 (81.04)	98 (18.96)	517 (81.03)
Yes	82 (67.77)	39 (32.23)	121 (18.97)
Congenital anomalies			
No	475 (77.74)	136 (22.26)	611 (95.77)
Yes	26 (96.30)	1 (3.70)	27 (4.23)
Blood transfusion			
No	458 (79.10)	121 (20.90)	579 (90.75)
One unit	33 (80.49)	8 (19.51)	41 (6.43)
Two unit	9 (60.00)	6 (40.00)	15 (2.35)
3 and above	1 (33.33)	2 (66.67)	3 (0.47)
Anemia			
No	455 (79.13)	120 (20.87)	575 (90.13)
Yes	46 (73.02)	17 (26.98)	63 (9.87)
Breastfeeding initiation			
No	434(77.64)	125(22.36)	559(87.62)
Yes	67(84.81)	12(15.19)	79(12.38)

^a^ APGAR, appearance, pulse, grimace, activity, and respiration; PNA, perinatal asphyxia; RDS, respiratory distress syndrome.

### 3.5 Causes of neonatal death

As shown in the table below, prematurity (34.31%) was the leading cause of neonatal death, followed by birth asphyxia, which accounted for 26.28% of neonatal deaths, whereas anemia was the least common cause of neonatal death (Table 5).

**Table 5:** Causes of neonatal death among neonates admitted to the NICU in the study.

Cause of death	Number	Percentage
Prematurity	47	34.31
Prenatal asphyxia	36	26.28
Sepsis	29	21.17
RDS^a^^,b^	13	9.49
Jaundice	3	2.19
Anemia	9	6.57
Total	137	100

^a^ RDS, respiratory distress syndrome.

^b^ Footnote: We acknowledge the relationship between prematurity and RDS as causes of death. RDS is primarily a disease of premature infants, and our study categorized deaths based on the primary cause as determined by the attending physician. While RDS primarily affects preterm infants, it can also affect term infants. Of the 245 preterm infants in our study, 63 (25.7%) had RDS listed as a primary cause of death, while 23 (5.9%) of the 393 term infants had RDS as a primary cause.

### 3.6 Survival status of the neonates and incidence rate of neonatal mortality

The study included 638 neonates, resulting in 2,046 neonate days. The study period was from January 1, 2021, to April 20, 2023, with a follow-up period of 1–21 days. The neonates’ discharge statuses were as follows: 37.1% (237 neonates) recovered, 21.5% (137 neonates) died, 34.0% (217 neonates) were transferred, and 7.4% (47 neonates) left against medical advice. The main reasons for transfer were prematurity and low birth weight (<1,500 g) requiring specialized NICU care, uncontrolled seizures in asphyxiated neonates, severe jaundice needing intensive phototherapy, respiratory distress (especially RDS) requiring CPAP, and severe sepsis. The total incidence rate of neonatal mortality at the NICU of Bichena Primary Hospital was 66.96 (CI 56.64, 79.17) per 1,000 neonate days.

The estimated cumulative survival probability during the follow-up period was 92.88% (95% CI: 90.46%–94.71%) on the first day, 84.33% (95% CI: 80.83%–87.24%) at the end of the second day, 77.35% (95% CI: 73.08%–81.03%) at the end of the third day, 59.73% (95% CI: 53.27%–65.60%) from days four to seven, and 55.61% (95%: 45.41%–64.66%) from days eight to 28 (Table 6).

**Table 6:** Life table analysis of neonatal survival among NICU admissions in the study.

Interval (only lower bound inclusive)	Beg. Total	Deaths	Lost^a^	Survival	Error	[95% Conf. Int.]
1- 2 days	638	41	124	0.9288	0.0107	0.9046 0.9471
2- 3 days	473	39	99	0.8433	0.0163	0.8083 0.8724
3- 4 days	335	24	90	0.7735	0.0202	0.7308 0.8103
4- 8 days	221	32	161	0.5973	0.0315	0.5327 0.6560
8- 28 days	28	1	27	0.5561	0.0494	0.4541 0.6466

^a^ Lost: when neonates were discharged, transferred to another facility, or left-against medical advice.

### 3.7 Kaplan–Meier and log-rank test results for several predictor variables

**Figure 1:**
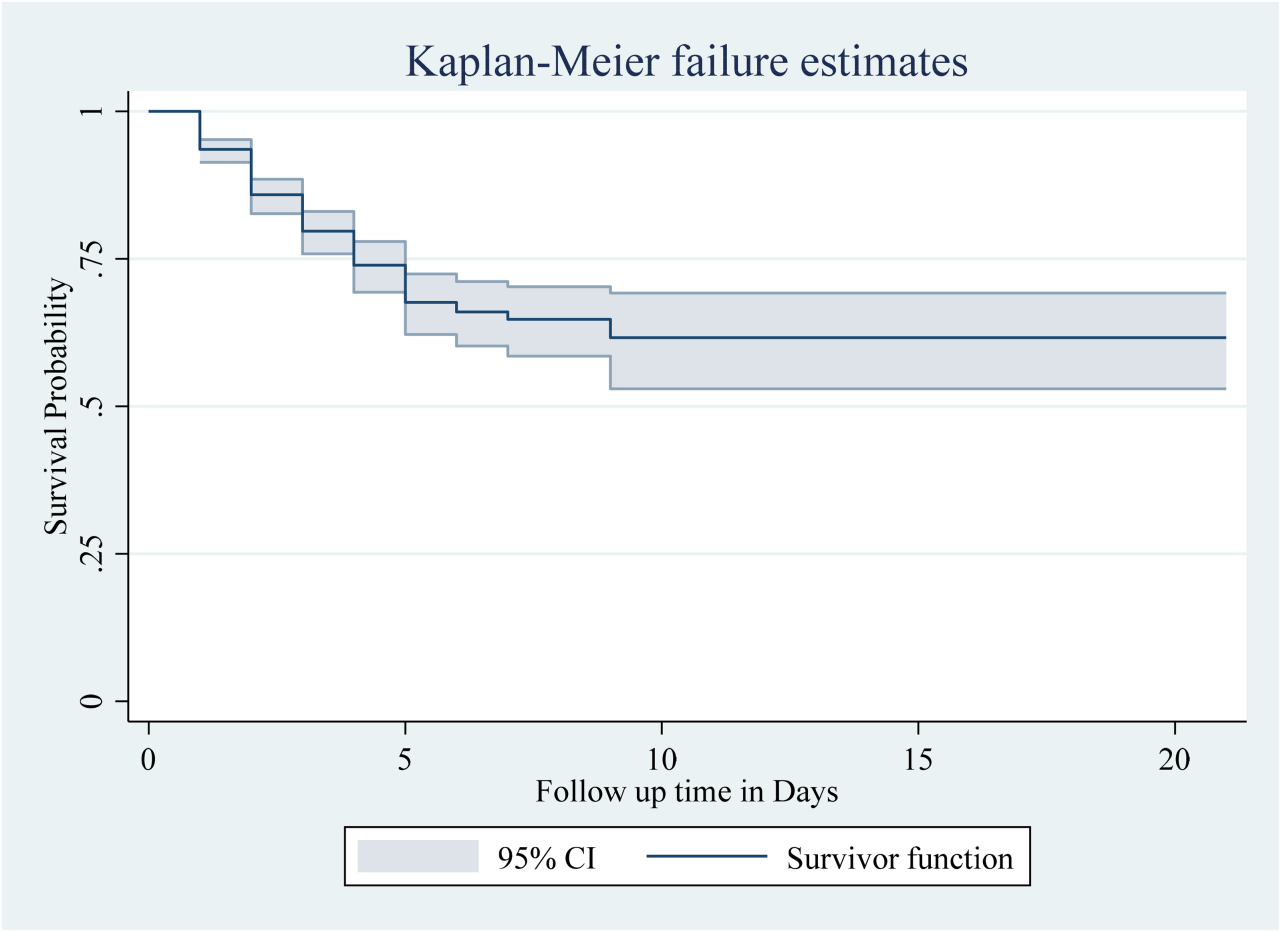
The overall Kaplan–Meier survival curve with a 95% confidence interval showing the survival time of neonates in the study.

The Kaplan–Meier survival estimate revealed that the survival probability of neonates was high on the first day after admission. However, this probability decreased over time. According to the graph, nearly all newborn deaths occurred within the first week of hospitalization. The total neonatal survival rate was 61.65%, with a standard error of 0.0416 (95% CI 0.52, 0.69) during the follow-up period (Figure 1).

**Figure 2:**
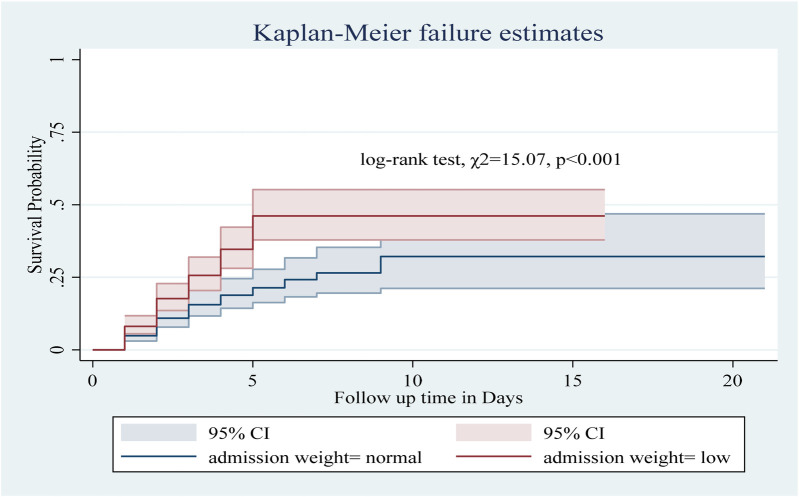
Kaplan–Meier curve showing the survival probability of neonates admitted to the NICU based on admission weight.

**Figure 3:**
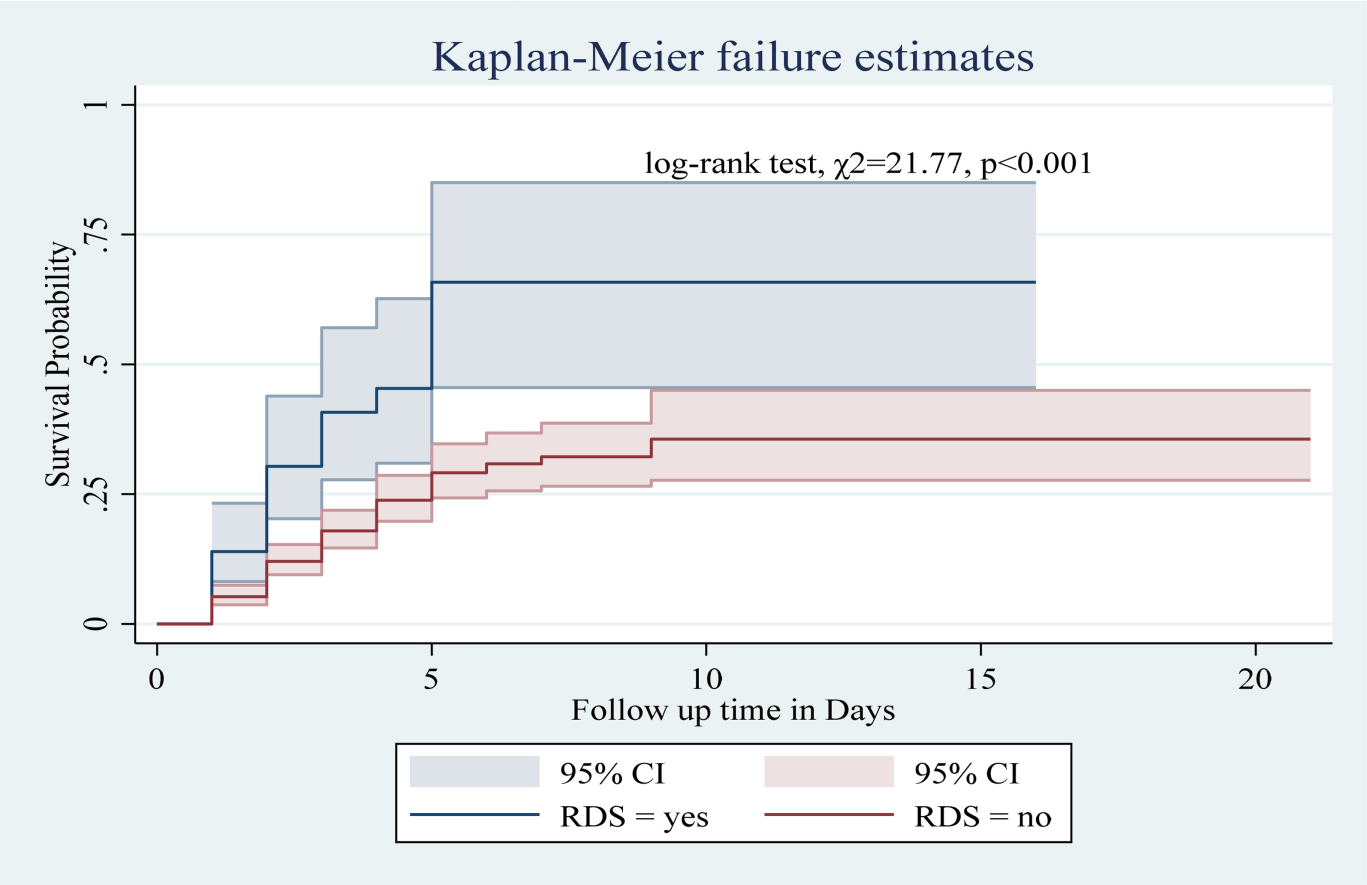
Kaplan–Meier curve showing the survival probability of neonates admitted to the NICU based on RDS.

**Figure 4:**
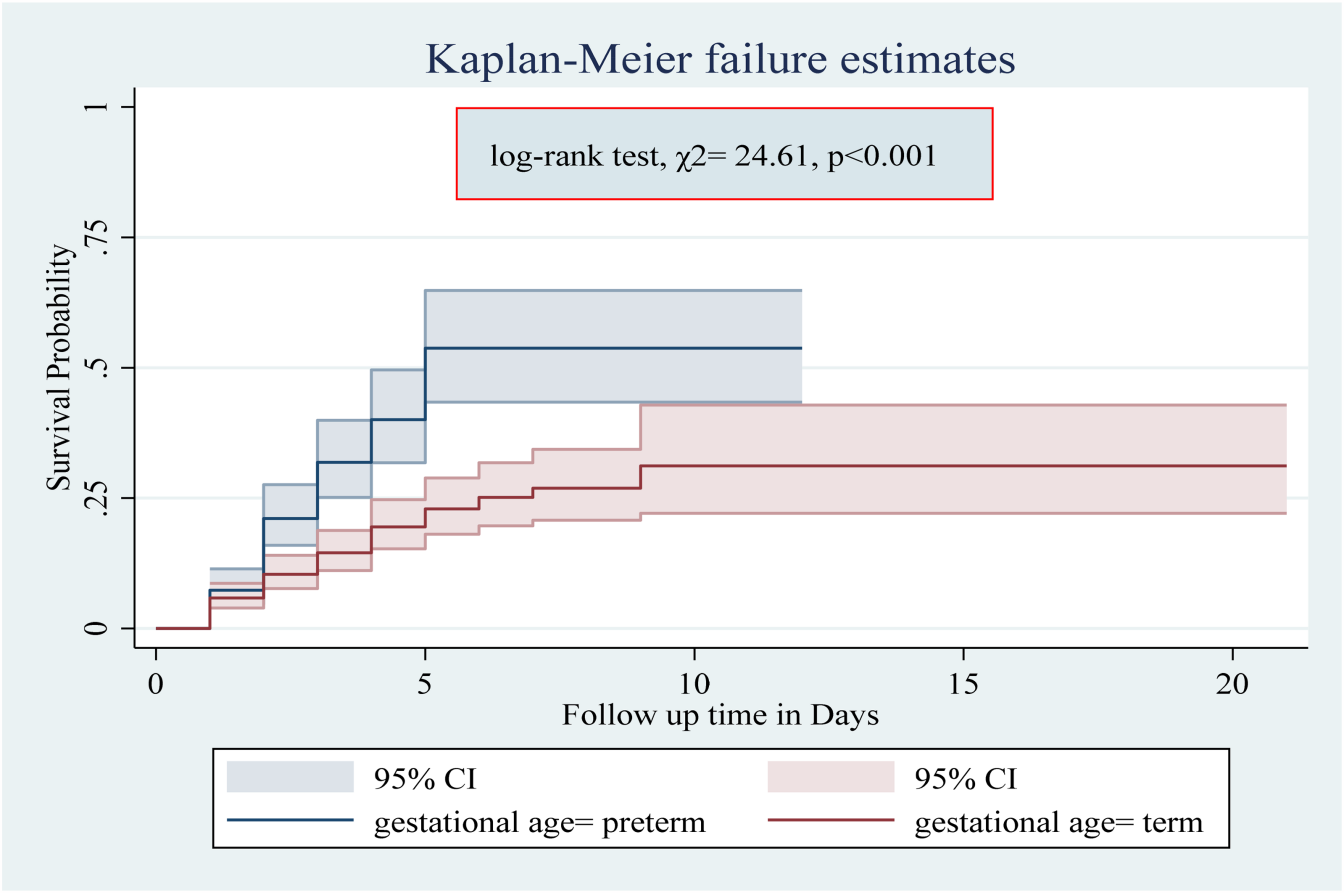
Kaplan–Meier curve showing the survival probability of neonates admitted to the NICU based on gestational age.

**Figure 5:**
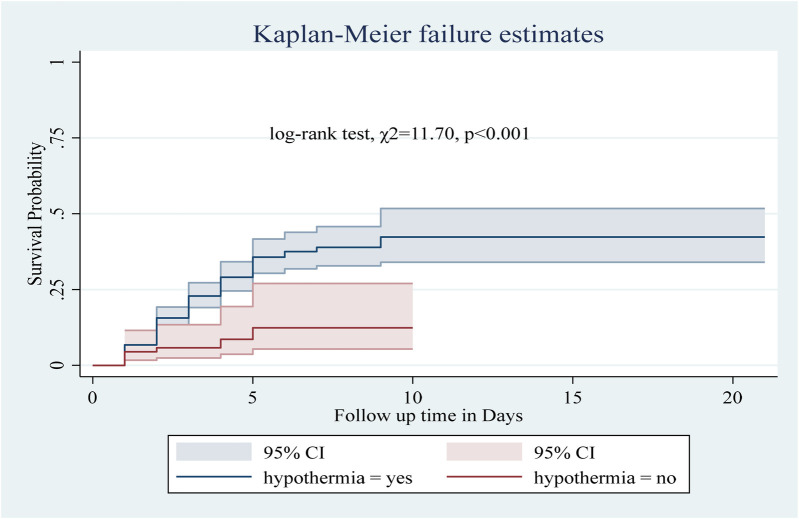
Kaplan–Meier curve showing the survival probability of neonates admitted to the NICU based on hypothermia.

**Figure 6:**
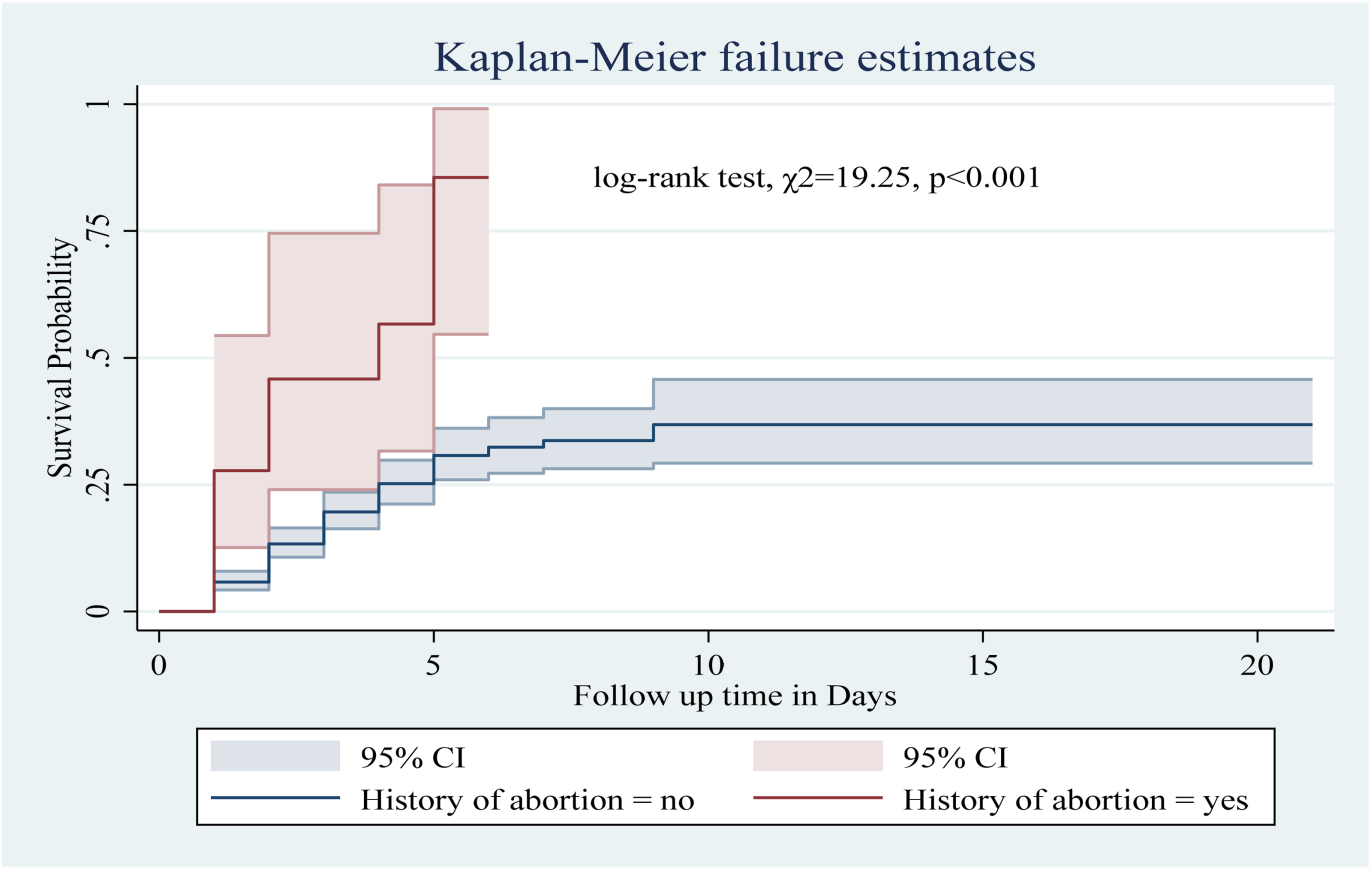
Kaplan–Meier curve showing the survival probability of neonates admitted to the NICU based on history of abortion.

Among the numerous predictor variables, log-rank tests revealed statistically significant differences in newborn survival probabilities. A lower birth weight (log-rank test, *χ*2 = 15.07, *p* < 0.001; Figure 2), respiratory distress (log-rank test, *χ*2 = 21.77, *p* < 0.001; Figure 3), preterm gestational age (log-rank test, *χ*2 = 24.61, *p* < 0.001; Figure 4), hypothermia (log-rank test, *χ*2 = 11.70, *p* < 0.001; Figure 5), and a history of abortion in a previous pregnancy (log-rank test, *χ*2 = 19.25, *p* < 0.001; Figure 6) were associated with a significantly lower probability of survival.

### 3.8 Predictors of neonatal mortality among neonates admitted to the NICU

In this study, bivariate Cox regression analysis was initially conducted with several variables, including hypothermia, birth injury/trauma, perinatal asphyxia, gestational age, history of abortion, birth weight, gravidity, premature ROM, prolonged ROM, suspected sepsis, amniotic fluid status, hepatitis exposure, residence, antepartum hemorrhage, and hypoglycemia. Variables showing a significance level of *p* < 0.25 in the bivariate analysis were further considered for the multivariable Cox regression model. The backward elimination method was employed for variable selection, with biological plausibility and insights from previous studies guiding the process. The final adjusted hazard ratios (AHRs) with 95% confidence intervals (CIs) were determined, and variables with a *p*-value < 0.05 were identified as significant predictors of neonatal mortality.

In this study, neonates with hypothermia had a 2.59-fold greater risk of death than those without hypothermia (AHR: 2.59, 95% CI: 1.17–5.70). Similarly, the presence of birth injury/trauma and perinatal asphyxia increased the risk of death by 1.82 (AHR: 1.82, 95% CI: 1.2, 2.76) and 1.65 times (AHR: 1.65, 95% CI: 1.11–2.45), respectively. Preterm neonates were at a 2.29-fold greater risk of death than term neonates (AHR: 2.29, 95% CI: 1.55, 3.38). Neonates from mothers with a history of abortion had a 2.13 times greater risk of death than those from mothers without a history of abortion (AHR: 2.13, 95% CI: 1.06, 4.25). Low birth weight increased the risk of death by 1.6 times (AHR: 1.6, 95% CI: 1.09, 2.34). Finally, neonates with hypoglycemia had a 1.71-fold greater risk of death than those without hypoglycemia (AHR: 1.71, 95% CI: 1.06 2.74) (Table 7).

**Table 7:** Bivariate and multivariate Cox regression analyses of predictive factors of neonatal mortality in the NICU.

Variables	Neonatal Outcomes		CHR(95%CI)	AHR(95%CI)	*P* value
	Censored	Death			
Residence					
Urban	291	66	1	1	
Rural	210	71	1.39 (1.00–1.95)	1.31 (0.93–1.85)	0.127
Birth weight					
Normal	275	54	1	1	
Low	226	83	1.91 (1.35–2.69)	1.6 (1.09–2.34)	0.016*
Gestational age					
Preterm	176	69	2.24 (1.60–3.14	2.29 (1.55–3.38)	0.000*
Term	325	68	1	1	
Hypothermia					
Yes	419	130	3.34 (1.56–7.15)	2.59 (1.17–5.70)	0.018*
No	82	7	1	1	
Perinatal Asphyxia					
No	396	78	1	1	
Yes	105	59	1.61 (1.14–2.26)	1.65 (1.11–2.45)	0.014*
Suspected sepsis					
Yes	327	104	1	1	
No	174	33	0.77 (0.52–1.14)	0.7 (0.46–1.06)	0.09
Amniotic fluid status					
Clear	417	99	1	1	
Meconium stained	84	38	1.30 (0.89–1.89)	1.26 (0.82–1.94)	0.296
Birth injury/trauma					
No	419	98	1	1	
Yes	82	39	1.59 (1.09- 2.30)	1.82 (1.2–2.76)	0.005*
Hypoglycemia					
No	461	115	1	1	
Yes	40	22	1.94 (1.23–3.06)	1.71 (1.06–2.74)	0.027*
History of abortion					
No	493	127	1	1	
Yes	8	10	3.67 (1.92–7.00)	2.13 (1.06–4.25)	0.033*
Gravidity					
Primigravida	242	56	1	1	
Multigravida	259	81	1.48 (1.05–2.08)	1.37 (0.95–1.97)	0.094
Premature ROM					
Yes	35	13	1	1	
No	466	124	0.55 (0.31–0.98)	0.6 (0.32–1.13)	0.115
Prolonged ROM					
No	484	127	1	1	
Yes	17	10	2.40 (1.26–4.57)	1.91 (0.94–3.89)	0.072
Hepatitis exposure					
Yes	6	3	1	1	
No	495	134	0.47 (0.15–1.49)	0.4 (0.12–1.36)	0.142
Antepartum hemorrhage					
Yes	4	2	1	1	
No	497	135	0.39 (0.10–1.60)	0.5 (0.12–2.13	0.348

ROM, rupture of membrane.

* Variables with *P* values <0.05 in the multivariable Cox regression analysis.

## 4 Discussion

The NMR at the NICU of Bichena Primary Hospital was 66.96 per 1,000 neonate days, significantly higher than rates reported in other regions of Ethiopia. For instance, Bombe Primary Hospital in southern Ethiopia reported a rate of 20.8 per 1,000 neonatal days (31), and Arba Minch General Hospital reported 31.6 per 1,000 neonate days of follow-up (28). Further, a study in Gurage Zone Public Hospitals reported 36.9 per 1,000 neonate days (32), and a tertiary hospital in Addis Ababa Ethiopia also reported 19.2 deaths per 1,000 live births (29). Such discrepancies can be attributed to differences in healthcare practices, hospital levels, sample size, and the demographic characteristics of admitted neonates. For example, Bombe Primary Hospital admitted a higher proportion of low-risk neonates, which may partly explain their lower NMR (31).

The NMR observed in our study was also much higher than international neonatal mortality rates, particularly in high-income and middle-income countries. In Australia, the NMR is approximately 2.3 per 1,000 live births, which is far lower than the rate observed in our study. Similarly, in middle-income countries, neonatal mortality remains significantly lower, with India reporting approximately 22.73 per 1,000 live births and South Africa around 11 per 1,000 live births. While our study reported NMR per 1,000 neonate days, these international figures, measured per 1,000 live births, still highlight major disparities in neonatal care (33).

Additionally, the study revealed a cumulative incidence rate of 215 per 1,000 newborns admitted to Bichena Primary Hospital, indicating that 21.5% of admitted newborns died during the follow-up period. This is twice the neonatal death rate at Bombe Primary Hospital, 10.6% (31) but comparable to rates at Arba Minch General Hospital (20.8%) (28), Debre Markos Hospital (21.3%) (34), and Gurage Zone Public Hospitals (22.7%) (32).

In this study, we identified several risk factors contributing to neonatal mortality. Being preterm increases the risk of death by 2.29 times compared with term neonates. This finding is supported by studies conducted in various locations, including Addis Ababa, Ethiopia (29), the Hadiya Zone (35), Jimma (36), and Debre Tabor (37). It is well known that preterm neonates are not fully prepared to live outside the uterus, as their bodies are not fully mature (29). Given the high risk of mortality and morbidity associated with preterm birth, it is better to transfer preterm neonates to Level II or Level III hospitals as soon as possible after stabilization for specialized care. Ensuring CPAP availability in primary hospitals is also crucial for improving respiratory support and reducing mortality in preterm neonates. Therefore, policymakers should prioritize CPAP provision and implement policies to facilitate prompt referrals to higher-level facilities, ultimately enhancing neonatal survival and long-term health outcomes.

Additionally, low-birth-weight neonates were at a 1.6-fold greater risk of death. This is in agreement with studies from Bombe (31), the Hadiya Zone (35), the Amhara regional state (38), Addis Ababa (39), and Asmara (10). Policymakers should prioritize establishing and maintaining a Level II or III NICU in hospitals that care for neonates, particularly in regions with high rates of preterm birth and low-birth-weight neonates. This will enable healthcare providers to provide specialized care and treatment to neonates who require it, and improve their chances of survival and long-term health outcomes. Additionally, hospitals should be equipped with the minimum set of equipment and supplies available to provide the most possible care for newborns, as per international standards and guidelines.

Similarly, this study revealed that perinatal asphyxia increased the risk of death by 1.65 times. This finding agrees with studies conducted in Hosanna (30), at a tertiary hospital, Addis Ababa (29), and Wolaita Sodo University Hospital in Ethiopia (40). A meta-analysis revealed that asphyxia increases the risk of neonatal death (41). Perinatal asphyxia leads to oxygen deprivation, which causes hypoxia, hypercarbia, and acidosis, potentially damaging vital organs and resulting in death (32). The unchanged death rate related to perinatal asphyxia may be attributed to delayed or inadequate care during labor and neonatal resuscitation (35). Consequently, pregnant women need to seek prompt medical care for any delivery complications. Clinicians should focus on providing timely, quality care, in early detection and management of perinatal asphyxia. Policymakers should prioritize improving maternal healthcare access by ensuring obstetricians are available during labor.

Furthermore, Neonates with hypothermia had a 2.59-fold greater risk of death than did those without hypothermia. This finding is consistent with studies conducted in Bombe primary hospitals (31), Wollega University referral hospitals (42), specialized hospitals in the Amhara region, and (38) the Hadiya Zone (35) from Ethiopia. A study from Malawi Hospital also reported that all neonates admitted with hypothermia died (43), indicating its significant role in mortality. This finding highlights the importance of keeping newborns warm and comfortable, especially in the first 24 h after birth. Clinicians should be alert about preventing neonatal hypothermia by using warm blankets and maintaining a cozy environment. Policymakers should ensure hospitals have access to warm blankets and heating devices to keep newborns safe and comfortable.

Moreover, this study revealed that birth injury/trauma increased the risk of death by 1.82 times. This finding is supported by studies conducted in Ethiopia (44). This might be related to cranial injuries in neonates, which can result in bleeding, hypovolemic shock, and various complications. These conditions deteriorate the health status of the neonate, resulting in neonatal death. This finding highlights the importance of careful monitoring and management of neonates with birth trauma, as these injuries may lead to adverse outcomes. Policymakers are recommended to improve access to quality maternal healthcare by training providers in emergency obstetric care and developing standards to minimize birth injuries.

Furthermore, hypoglycemia increases the risk of death by 1.71 times compared with those without this condition. A study conducted in Ethiopia supports the present study (38). The possible reason may be a delay in the diagnosis of hypoglycemia, as undiagnosed and untreated hypoglycemia can cause brain damage and potentially result in death (25). This finding emphasizes the importance of early diagnosis and treatment of hypoglycemia in neonates. Parents should be aware of the signs and symptoms, such as shakiness, irritability, and lethargy, and seek medical attention if they suspect hypoglycemia. For sick neonates unable to breastfeed, alternative feeding methods such as cup or tube feeding should be considered, particularly during transport, to help maintain glucose levels. Clinicians also should monitor blood glucose levels and provide supplements as needed.

Finally, neonates from mothers with a history of abortion had a 2.13-fold greater risk of death than those from mothers without a history of abortion. This finding aligns with those of previous studies in Ethiopia and abroad (45–48). Further research is needed to explore the biological mechanism of different types of abortion-related to subsequent pregnancy (49). This finding emphasizes the importance of pregnant women disclosing previous abortions and reproductive health issues to their healthcare providers. Clinicians should be aware of abortion history as a risk factor and take steps to identify and manage underlying health issues. Policymakers should improve access to quality reproductive healthcare through education and counseling on reproductive health and family planning.

This study has several strengths, including a large sample size of 638 neonates, a cohort study design, and standardized data collection by trained NICU nurses. The study accounted for seasonal variation over two years and provides valuable insights for future prospective cohort studies in primary hospitals. However, the study also has some limitations. The retrospective design did not address potential predictors such as socioeconomic status, quality of care, or the service provider. Additionally, the study was conducted in a single health institution and had potential loss to follow-up. Moreover, the absence of CPAP in the NICU may have impacted neonatal outcomes. Addressing these limitations and focusing on identified risk factors can help future research improve neonatal care and outcomes.

## 5 Conclusion

In this study, we reported a high rate of neonatal mortality, exceeding rates reported in other regions of Ethiopia. Hypothermia, birth injury, perinatal asphyxia, preterm birth, maternal history of abortion, low birth weight, and neonatal hypoglycemia were independent predictors of neonatal mortality. As our findings revealed, most predictors were preventable and treatable. Therefore, special attention should be given to the early identification of obstetric complications, immediate interventions, and postnatal care to reduce the risk of neonatal mortality and enhance overall neonatal outcomes.

## Data Availability

The raw data supporting the conclusions of this article will be made available by the authors, without undue reservation.
